# Similar burden of rare genetic variants in ischemic and non-ischemic dilated cardiomyopathy

**DOI:** 10.3389/fcvm.2025.1542653

**Published:** 2025-04-29

**Authors:** Louie Cao, Joshua Rushakoff, Ian Williamson, Anja Karlstaedt, Michelle Kittleson, Lawrence Czer, Evan P. Kransdorf

**Affiliations:** Smidt Heart Institute, Cedars-Sinai Medical Center, Los Angeles, CA, United States

**Keywords:** ischemic cardiomyopathy, dilated cardiomyopathy, heart transplantation, genetic testing, ischemic dilated cardiomyopathy

## Abstract

**Background:**

The aim of the study was to determine the prevalence of rare disease-causing variants in cardiomyopathy-associated genes in a cohort of patients with ischemic and non-ischemic dilated cardiomyopathy undergoing heart transplant.

**Methods:**

We conducted a single-center cohort study of 60 adult patients with left ventricular ejection fraction ≤50% and left ventricular end-diastolic dimension ≥95th percentile for sex/height who underwent heart transplant between January 2017 and December 2023 and consented to participate in a cardiac tissue biobank. We evaluated the prevalence of rare (minor allele frequency <0.1%) disease-causing (pathogenic or likely pathogenic by American College of Genetics and Genomics criteria) variants in cardiomyopathy-associated genes.

**Results:**

A total of 60 individuals fulfilled the inclusion criteria: 16 with ischemic dilated cardiomyopathy [88% men, median age 65 years, interquartile range (IQR) 64–68 years] and 44 with non-ischemic dilated cardiomyopathy (80% men, median age 53 years, IQR 39–65 years). We found that the prevalence of disease-causing variants was similar between patients with ischemic dilated cardiomyopathy (3/16 or 19%; 95% credible interval 6%–36%) and those with non-ischemic dilated cardiomyopathy (10/44 or 23%; 95% credible interval 12%–33%). Variants in the ischemic dilated cardiomyopathy group were found in the *TTN* and *DMD* genes. Variants in the non-ischemic dilated cardiomyopathy group were found in the *TTN*, *FLNC*, *LMNA*, *MYH7*, and *RBM20* genes.

**Conclusions:**

Patients with ischemic dilated cardiomyopathy undergoing heart transplant possessed a similar burden of rare disease-causing variants as those with non-ischemic dilated cardiomyopathy. Our results suggest that genetic testing may be beneficial in patients with advanced heart failure requiring heart transplant due to ischemic dilated cardiomyopathy to detect disease-causing variants in cardiomyopathy-associated genes.

## Background

Ischemic and non-ischemic cardiomyopathy are the most common indications for heart transplantation, comprising approximately 30% and 50% of heart transplants in both Europe and the United States, respectively (1). Although definitions vary, a standardized definition for ischemic cardiomyopathy is symptomatic heart failure with left ventricular ejection fraction (LVEF) ≤40% and a history of myocardial infarction or revascularization, ≥75% stenosis of the left main or proximal left anterior descending coronary arteries, or ≥75% stenosis of at least two epicardial arteries (2). In contrast, non-ischemic dilated cardiomyopathy (NIDCM) is defined as LVEF ≤50% and left ventricular end-diastolic diameter (LVEDD) ≥95th percentile for sex and height while excluding coronary artery disease (CAD), abnormal loading conditions, or cardiac toxins (3).

Imaging studies suggest that ischemic cardiomyopathy results from acute ischemic injury to the myocardium and that the severity of the resulting left ventricular dysfunction is proportional to the degree of ischemic injury (4, 5). The pathophysiology of non-ischemic dilated cardiomyopathy is generally considered to be distinct, and rare disease-causing genetic variants, those classified by the American College of Genetics and Genomics criteria as likely pathogenic or pathogenic (LP/P), can be identified in 20% of patients (6, 7). However, a subset of patients with ischemic cardiomyopathy develops ventricular dilation, referred to as ischemic dilated cardiomyopathy (IDCM). It is difficult to distinguish IDCM from NIDCM because patients can have risk factors or features of both diseases. When evaluated by cardiac magnetic resonance imaging (CMRI), patients with IDCM and DCM exhibit overlap in ventricular volume and late gadolinium enhancement patterns (8). These observations suggest that IDCM and NIDCM may share pathophysiologic features.

We hypothesized that patients with IDCM harbor disease-causing variants in cardiomyopathy-associated genes at a prevalence similar to that of patients with NIDCM. Thus, we sought to determine the prevalence of rare disease-causing variants in cardiomyopathy-associated genes in a cohort of patients with IDCM and NIDCM undergoing heart transplant. Therefore, this study tests our hypothesis that rare disease-causing genetic variants contribute to the development of advanced heart failure due to IDCM.

## Methods

### Study cohort

**Figure 1:**
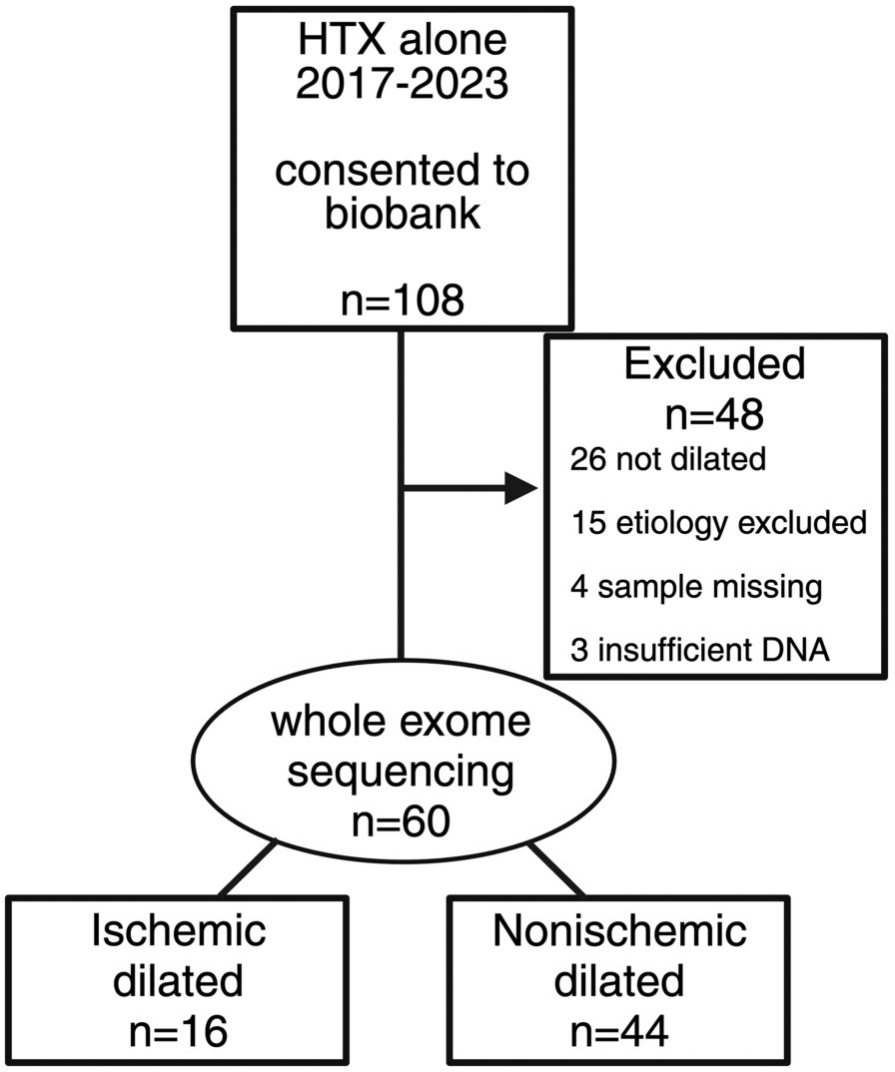
CONSORT diagram of patient flow for the study cohort. A total of 108 patients underwent heart transplant alone between 1 January 2017 and 31 December 2023 who were consented to a cardiac tissue biobank. Of them, 48 patients were excluded for reasons indicated in the figure. Whole exome sequencing was performed on 60 patients, 16 with ischemic dilated cardiomyopathy and 44 with non-ischemic dilated cardiomyopathy.

We identified 60 patients who consented to participate in a cardiac tissue biobank (Cedars-Sinai IRB protocols Pro00010979 and Study00001617) with dilated cardiomyopathy [defined as LVEF ≤ 50% and LVEDD ≥ 95th percentile for sex and height (3)] and underwent heart transplant at Cedars-Sinai Medical Center in Los Angeles, California, between 1 January 2017 and 31 December 2023 for ischemic or non-ischemic cardiomyopathy. Patients with other forms of cardiomyopathy, including amyloidosis, sarcoidosis, and peripartum, were excluded (Figure 1). Patients with a history of myocardial infarction or revascularization, ≥75% stenosis of the left main or proximal left anterior descending coronary arteries, or ≥75% stenosis of at least two epicardial arteries were classified as having IDCM per the Felker definition (2). Echocardiography was performed after coronary revascularization but before heart transplantation.

### Exome sequencing, gene selection, variant filtering, and variant interpretation

For a full description of methods, see the online Supplementary Material. Briefly, genomic DNA was isolated from explanted hearts for whole exome sequencing. We assessed for variants in 36 genes associated with DCM, as previously selected in the DCM Precision Medicine Study (7) and the DMD given its association with DCM (9, 10). Variants were assigned as benign/likely benign or LP/P if the variant had that classification in ClinVar with a two-star rating or above. For variants not present in ClinVar, variant interpretation was performed using the ClinGen guidelines for DCM (11) with modifications from the DCM Precision Medicine Study (7). For variants in *TTN*, criterion PVS1_strong was only applied if the variants were in the A-band or an exon with percent splice in >90%. The variant interpretation criteria we used are detailed in the online Supplementary Material.

### Statistical analysis

Statistical analysis was performed using R version 4.2.2 (12). Variables were compared using the Kruskal–Wallis test (non-Gaussian continuous variables), *t*-test (Gaussian continuous variables), and the chi-square test (categorical variables). A power analysis was performed using the R package “pwrss” (13), which revealed that our study cohort size (*n* = 60) provided a power of 0.51 to detect a twofold difference in the odds of a patient in the IDCM group possessing a disease-causing variant (given a baseline probability of 0.2 and type I error rate of 0.05). Therefore, we used Bayesian methods implemented in the R packages “BayesFactor” (14) and “bayestestR” (15) to calculate the 95% credible interval of the proportion of patients in each group carrying a disease-carrying variant.

## Results

Of the 60 patients with dilated cardiomyopathy, 16 were classified as IDCM (27%) and 44 as NIDCM (73%). Patients with IDCM were older (median age 65 vs 53 years, IQR 64–68 vs. 39–65 years; *p* < 0.001) but similar in sex, race, ethnicity, and body mass index (Table 1). Patients with IDCM more frequently had diabetes mellitus (81% vs. 23%; *p* < 0.001) and hyperlipidemia (100% vs. 41%; *p* < 0.001). There was no difference between patients with IDCM and NIDCM with regard to the frequency of myocarditis, aortic or mitral valve disease requiring replacement, atrial or ventricular arrhythmias, or the presence of a cardiac implantable electronic device (Table 2). Most patients with IDCM had a history of coronary revascularization (94% vs. 0%; *p* < 0.001) or myocardial infarction (94% vs. 0%; *p* < 0.001). There was no difference between patients with IDCM and NIDCM with regard to a family history of cardiomyopathy or sudden cardiac arrest. Finally, there was no difference in mean left ventricular ejection fraction (17% vs. 17%; *p* = 0.849) or mean LVEDD (7.1 vs. 7.1 cm; *p* = 0.733) between the groups on the echocardiogram performed closest before transplant.

**Table 1:** Characteristics of patients in the study cohort with ischemic dilated versus non-ischemic dilated cardiomyopathy.

Factor	IDCM *n* = 16	NIDCM *N* = 44	*P*-value
Age at HTX	65 [64–68]	53 [39–65]	<0.001
Male	14 (88%)	35 (79.5%)	0.710
Race			0.091
White	11 (69%)	24 (55%)	
Black	0	10 (23%)	
Asian/Other	5 (31%)	10 (23%)	
Hispanic	5 (31%)	14 (32%)	1.000
BMI	26 [24–28]	26 [23–28]	0.828

BMI, body mass index.

Age and BMI are presented as mean [interquartile range].

**Table 2:** Medical history of patients in the study cohort with ischemic dilated versus non-ischemic dilated cardiomyopathy.

Factor	IDCM *n* = 16	NIDCM *N* = 44	*P*-value
Medical history			
Heavy alcohol use	0	3 (7%, 0%–14%)	0.558
Diabetes mellitus	13 (81%, 62%–100%)	10 (23%, 10%–35%)	<0.001
Hyperlipidemia	16 (100%)	18 (41%, 26%–55%)	<0.001
Hx myocarditis	0	5 (11%, 2%–21%)	0.311
Hx MVR	4 (25%, 4%–46%)	4 (9%, 1%–18%)	0.192
Hx AVR	0	4 (9%, 1%–18%)	0.565
Hx atrial arrhythmias	9 (56%, 32%–81%)	28 (64%, 49%–78%)	0.826
Hx ventricular arrhythmias	8 (50%, 26%–74%)	24 (55%, 40%–69%)	0.984
CIED	12 (75%, 54%–96%)	32 (73%, 60%–86%)	1.000
Hx PCI or CABG	15 (94%, 82%–100%)	0	<0.001
Hx MI	15 (94%, 82%–100%)	0	<0.001
Family history of CM	4 (25%, 4%–46%)	18 (41%, 26%–55%)	0.408
Family history of SCD	0	2 (5%, 0%–11%)	1.000
Echocardiography			
EF	16.4 (5.43)	16.7 (5.65)	0.849
LVEDD	7.12 (0.65)	7.06 (0.73)	0.733

AVR, aortic valve replacement; CABG, coronary artery bypass grafting; CIED, cardiac implantable electronic device; CM, cardiomyopathy; EF, ejection fraction; Hx, history; LVEDD, left ventricular end-diastolic diameter; MI, history of myocardial infarction; MVR, mitral valve replacement; PCI, percutaneous coronary intervention; SCD, sudden cardiac death; SVT, supraventricular tachycardia; VF, ventricular fibrillation; VT, ventricular tachycardia.

(1) Medical history variables are presented as number (proportion, 95% confidence intervals). (2) Echocardiographic variables EF and LVEDD are presented as mean (standard deviation).

**Figure 2:**
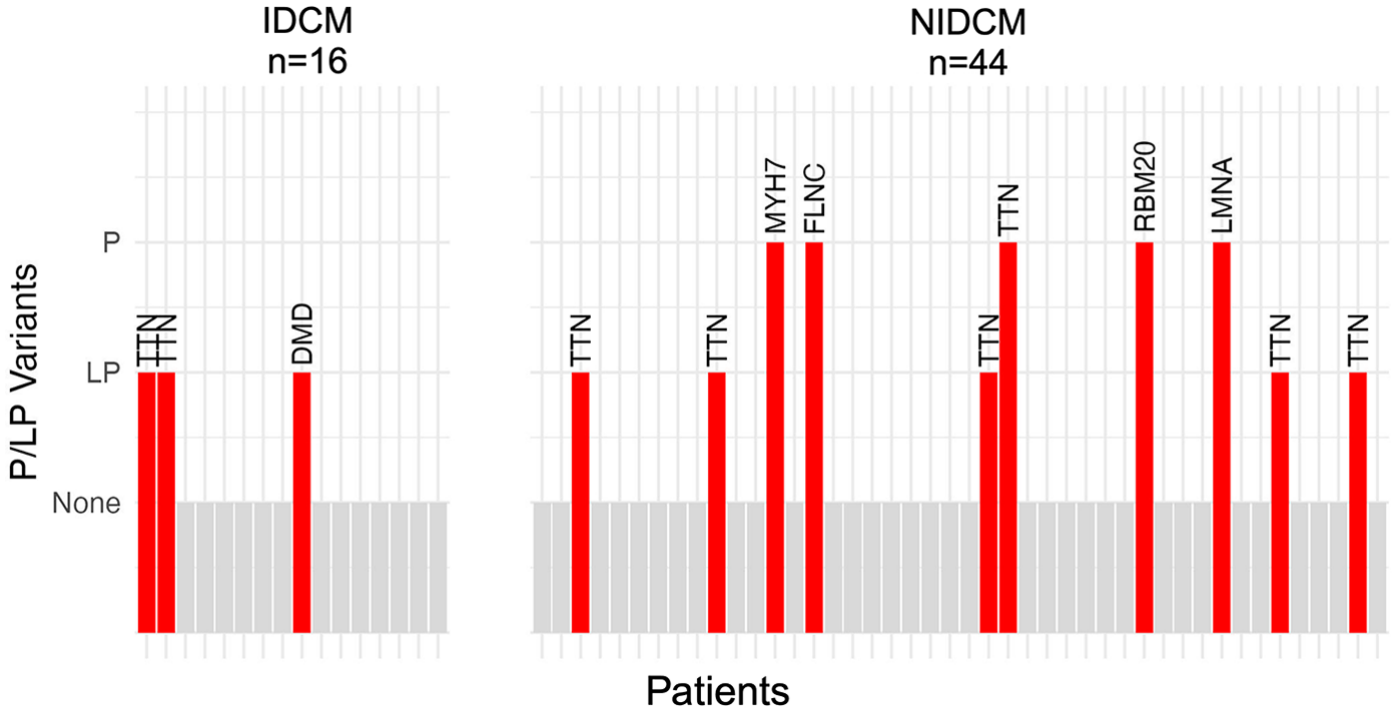
Bar plot of patients with rare disease-causing (likely pathogenic or pathogenic) variants in the study cohort. Each patient is depicted by a vertical bar; patients without a disease-causing variant are depicted by gray bars and patients with a disease-causing variant are depicted by red bars. The name of the gene in which the variant was found is indicated above the bar. The prevalence of disease-causing variants was similar between patients with ischemic dilated cardiomyopathy (bars on left; 3/16 or 19%; 95% credible interval 6%–36%) and those with non-ischemic dilated cardiomyopathy (bars on right; 10/44 or 23%; 95% credible interval 12%–33%).

We identified 13 P/LP variants in six cardiomyopathy-associated genes within the 60 patients in the study cohort (variants are described in Table 3 and Supplementary Table S1). Of the 13 variants, 10 were previously identified in patients with dilated cardiomyopathy (Supplementary Table S1) and three were novel. The proportion of patients with P/LP variants was similar between the groups, with 3/16 (19%; 95% credible interval 6%–36%) patients in the IDCM group and 10/44 (23%; 95% credible interval 12%–33%) patients in the NIDCM group (Figure 2). Most (8/13 or 62%) P/LP variants were found in *TTN*, with no difference in the proportion of patients with *TTN* variants between the IDCM (2/16 or 13%; 95% credible interval 3%–26%) and NIDCM groups (6/44 or 14%; 95% credible interval 5%–22%).

**Table 3:** Pathogenic/likely pathogenic variants identified in the study cohort.

Variant^a^	Gene	Coding	Protein	Class
Ischemic dilated cardiomyopathy				
chr2-178548829-GTT-G	TTN	ENST00000589042 c.92795_92796del	ENSP00000467141 p.Lys30932ThrfsTer6	LP
chr2-178577785-G-A	TTN	ENST00000589042 c.68641C>T	ENSP00000467141 p.Arg22881Ter	LP
chrX-32614303-CT-C	DMD	ENST00000357033 c.1481del	ENSP00000354923 p.Lys494ArgfsTer7	LP
Non-ischemic dilated cardiomyopathy				
chr1-156134838-C-T	LMNA	ENST00000368300 c.673C>T	ENSP00000357283 p.Arg225Ter	P
chr2-178547890-TG-T	TTN	ENST00000589042 c.93735del	ENSP00000467141 p.Val31247Ter	LP
chr2-178560772-GT-G	TTN	ENST00000589042 c.85359del	ENSP00000467141 p.Pro28454GlnfsTer7	LP
chr2-178567508-TC-T	TTN	ENST00000589042 c.78623del	ENSP00000467141 p.Gly26208GlufsTer21	LP
chr2-178570990-ATTCT-A	TTN	ENST00000589042 c.75138_75141del	ENSP00000467141 p.Lys25046AsnfsTer8	P/LP
chr2-178612453-CTCTTTTCCACAATG-C	TTN	ENST00000589042 c.50058_50071del	ENSP00000467141 p.Tyr16686Ter	LP
chr2-178738152-G-T	TTN	ENST00000589042 c.14301C>A	ENSP00000467141 p.Cys4767Ter	LP
chr7-128844045-C-T	FLNC	ENST00000325888 c.2971C>T	ENSP00000327145 p.Arg991Ter	P/LP
chr10-110812298-G-A	RBM20	ENST00000369519 c.1901G>A	ENSP00000358532 p.Arg634Gln	P/LP
chr14-23425970-C-T	MYH7	ENST00000355349 c.2156G>A	ENSP00000347507 p.Arg719Gln	P

LP, likely pathogenic; P, pathogenic.

^a^ Variants are described as chromosome-position-reference-alternate, with position provided per GRCh38.

## Discussion

In a cohort of patients with dilated cardiomyopathy who underwent heart transplant, patients with IDCM or NIDCM had a similar prevalence of disease-causing variants in cardiomyopathy-associated genes compared to NIDCM patients. Our results are consistent with previous studies suggesting a superimposed non-ischemic component in ischemic cardiomyopathy. Using cardiac magnetic resonance imaging, approximately 15% of patients with CAD have a superimposed non-ischemic scar pattern (16, 17). Disease-causing variants contribute to lower EF and worse outcomes in patients with CAD (16).

Two previous studies have investigated the interplay between CAD, heart failure, and disease-causing genetic variants in cardiomyopathy-associated genes. In 2021, Povysil et al. reported the identification of disease-causing variants in ICM patients from the Candesartan in Heart Failure-Assessment of Reduction in Mortality and Morbidity (CHARM) and Controlled Rosuvastatin Multinational Trial in Heart Failure (CORONA) clinical trials; however, the prevalence of disease-causing variants was substantially lower than the IDCM group in our study at 3% (18). More recently, Jones et al. reported the identification of disease-causing variants in cardiomyopathy-associated genes in patients with CAD in the UK Biobank, also with a lower prevalence of disease-causing variants than the IDCM group in our study at 0.6% (16). We identified disease-causing variants in 19% of the patients with IDCM in our cohort of patients with advanced heart failure requiring heart transplant. Although we utilized a larger panel of DCM-associated genes, *TTN* was the most prevalent gene with disease-causing variants in all three studies. We posit that the higher prevalence of disease-causing variants we observed in the IDCM group in our study may be due to the increased severity of left ventricular dysfunction, given that all patients in our study cohort had undergone heart transplant. Further prospective investigation is needed to test this hypothesis.

Genetic testing and cascade screening is recommended in NIDCM (19); however, no guidance exists on genetic testing in patients with IDCM. Our results suggest that it may be prudent to extend this recommendation to patients similar to our study cohort: those with advanced heart failure due to IDCM requiring heart transplant. Our findings may also explain why some patients with ICM do not benefit from revascularization (20–23), i.e., in these cases, extant left ventricular dilation and dysfunction may be driven, at least in part, by genetic mechanisms.

Although our study is limited by a small cohort size compared to heart failure clinical trials, a cohort of 60 heart transplant recipients is substantial and would represent 3–6 times the annual volume of most heart transplant centers in Europe and the United States, where the median volume is 10–20 heart transplants per year (1). Although women were underrepresented in our study cohort, it was ethnically diverse, and it was also representative with respect to the proportion of heart transplants due to ICM and NICM. We were only able to enroll a portion of patients undergoing heart transplantation at our center during the study period in the cardiac tissue biobank used for this study, yielding a potential selection bias. A second potential selection bias is that given that the study cohort was composed of patients with advanced heart failure requiring heart transplant, the frequency of disease-causing variants in the IDCM group may be higher than in patients with less severe cardiomyopathy.

## Conclusions

In a cohort of heart transplant recipients with DCM, we found a similar prevalence of rare disease-causing variants in cardiomyopathy-associated genes in patients with IDCM as compared to NIDCM. Our results suggest that genetic testing may be beneficial in patients with advanced heart failure requiring heart transplant due to IDCM to detect disease-causing variants in cardiomyopathy-associated genes.

## Data Availability

The datasets presented in this article are not readily available because the aforementioned IRB protocols did not provide for public release of genetic data. Requests to access the datasets can be directed to the corresponding author.

## References

[1] Khush KK Cherikh WS Chambers DC Harhay MO Hayes D Jr Hsich E The International Thoracic Organ Transplant Registry of the International Society for Heart and Lung Transplantation: thirty-sixth adult heart transplantation report - 2019; focus theme: donor and recipient size match. J Heart Lung Transplant. (2019) 38(10):1056–66. 10.1016/j.healun.2019.08.004 31548031 PMC6816343

[2] Felker GM Shaw LK O'Connor CM. A standardized definition of ischemic cardiomyopathy for use in clinical research. J Am Coll Cardiol. (2002) 39(2):210–8. 10.1016/S0735-1097(01)01738-7 11788209

[3] Kinnamon DD Morales A Bowen DJ Burke W Hershberger RE, Consortium* DCM. Toward genetics-driven early intervention in dilated cardiomyopathy: design and implementation of the DCM precision medicine study. Circ Cardiovasc Genet. (2017) 10(6):e001826. 10.1161/CIRCGENETICS.117.001826 29237686 PMC5842914

[4] Masci PG Ganame J Francone M Desmet W Lorenzoni V Iacucci I Relationship between location and size of myocardial infarction and their reciprocal influences on post-infarction left ventricular remodelling. Eur Heart J. (2011) 32(13):1640–8. 10.1093/eurheartj/ehr064 21398642

[5] Feistritzer HJ Nanos M Eitel I Jobs A de Waha-Thiele S Meyer-Saraei R Determinants and prognostic value of cardiac magnetic resonance imaging-derived infarct characteristics in non-ST-elevation myocardial infarction. Eur Heart J Cardiovasc Imaging. (2020) 21(1):67–76. 10.1093/ehjci/jez165 31518417

[6] Verdonschot JAJ Hazebroek MR Krapels IPC Henkens M Raafs A Wang P Implications of genetic testing in dilated cardiomyopathy. Circ Genom Precis Med. (2020) 13(5):476–87. 10.1161/CIRCGEN.120.003031 32880476

[7] Jordan E Kinnamon DD Haas GJ Hofmeyer M Kransdorf E Ewald GA Genetic architecture of dilated cardiomyopathy in individuals of African and European ancestry. JAMA. (2023) 330(5):432–41. 10.1001/jama.2023.11970 37526719 PMC10394581

[8] Won E Donnino R Srichai MB Sedlis SP Feit F Rolnitzky L Diagnostic accuracy of cardiac magnetic resonance imaging in the evaluation of newly diagnosed heart failure with reduced left ventricular ejection fraction. Am J Cardiol. (2015) 116(7):1082–7. 10.1016/j.amjcard.2015.06.032 26251006 PMC4567940

[9] Restrepo-Cordoba MA Wahbi K Florian AR Jimenez-Jaimez J Politano L Arad M Prevalence and clinical outcomes of dystrophin-associated dilated cardiomyopathy without severe skeletal myopathy. Eur J Heart Fail. (2021) 23(8):1276–86. 10.1002/ejhf.2250 34050592

[10] Johnson R Otway R Chin E Horvat C Ohanian M Wilcox JAL DMD-associated dilated cardiomyopathy: genotypes, phenotypes, and phenocopies. Circ Genom Precis Med. (2023) 16(5):421–30. 10.1161/CIRCGEN.123.004221 37671549 PMC10592075

[11] Morales A Kinnamon DD Platt JE Vatta J Dorschner M O M Variant interpretation for dilated cardiomyopathy: refinement of the American college of medical genetics and genomics/ClinGen guidelines for the DCM precision medicine study. Circ Genom Precis Med. (2020) 13(2):e002480. 10.1161/CIRCGEN.119.002480 32160020 PMC8070981

[12] R Core Team. R: A Language and Environment for Statistical Computing. R Foundation for Statistical Computing, Vienna, Austria (2023). Available at: https://www.R-project.org/ (Accessed March 20, 2025).

[13] Bulus M. pwrss: Statistical Power and Sample Size Calculation Tools. R package version 0.3.1 (2023). Available at: https://CRAN.R-project.org/package=pwrss (Accessed March 20, 2025).

[14] Morey R Rouder J. BayesFactor: Computation of Bayes Factors for Common Designs. R package version 0.9 (2023). Available at: https://richarddmorey.github.io/BayesFactor/ (Accessed March 20, 2025).

[15] Makowski D Ben-Shachar MS Lüdecke D Bayestest R. Describing effects and their uncertainty, existence and significance within the Bayesian framework. J Open Source Softw. (2019) 4(40):1541. 10.21105/joss.01541

[16] Jones RE Hammersley DJ Zheng S McGurk KA de Marvao A Theotokis PI Assessing the association between genetic and phenotypic features of dilated cardiomyopathy and outcome in patients with coronary artery disease. Eur J Heart Fail. (2024) 26(1):46–55. 10.1002/ejhf.3033 37702310 PMC11216513

[17] Bawaskar P Thomas N Ismail K Guo Y Chhikara S Athwal PSS Nonischemic or dual cardiomyopathy in patients with coronary artery disease. Circulation. (2024) 149(11):807–21. 10.1161/CIRCULATIONAHA.123.067032 37929565 PMC10951941

[18] Povysil G Chazara O Carss KJ Deevi SVV Wang Q Armisen J Assessing the role of rare genetic variation in patients with heart failure. JAMA Cardiol. (2021) 6(4):379–86. 10.1001/jamacardio.2020.6500 33326012 PMC7745141

[19] Hershberger RE Givertz MM Ho CY Judge DP Kantor PF McBride KL Genetic evaluation of cardiomyopathy: a clinical practice resource of the American College of Medical Genetics and Genomics (ACMG). Genet Med. (2018) 20(9):899–909. 10.1038/s41436-018-0039-z 29904160

[20] Velazquez EJ Lee KL Deja MA Jain A Sopko G Marchenko A Coronary-artery bypass surgery in patients with left ventricular dysfunction. N Engl J Med. (2011) 364(17):1607–16. 10.1056/NEJMoa1100356 21463150 PMC3415273

[21] Perera D Clayton T O'Kane PD Greenwood JP Weerackody R Ryan M Percutaneous revascularization for ischemic left ventricular dysfunction. N Engl J Med. (2022) 387(15):1351–60. 10.1056/NEJMoa2206606 36027563

[22] Iaconelli A Pellicori P Dolce P Busti M Ruggio A Aspromonte N Coronary revascularization for heart failure with coronary artery disease: a systematic review and meta-analysis of randomized trials. Eur J Heart Fail. (2023) 25(7):1094–104. 10.1002/ejhf.2911 37211964

[23] Moliner-Abos C Calvo-Barcelo M Sole-Gonzalez E Borrellas Martin A Fluvia-Brugues P Sanchez-Vega J Revascularization and outcomes in ischaemic left ventricular dysfunction after heart failure admission: the RevascHeart study. Eur J Heart Fail. (2025) 27(3):598–605. 10.1002/ejhf.3463 39359034

