# Supplementary material for: Similar burden of rare genetic variants in ischemic and non-ischemic dilated cardiomyopathy
Source: Front Cardiovasc Med. 2025 Apr 29;12:1542653. doi: 10.3389/fcvm.2025.1542653 (PMC12069280; doi:10.3389/fcvm.2025.1542653)
Supplement: Supplementary file 1 [file Datasheet1.docx]

**Online Supplementary Material for “Similar Burden of Rare Genetic Variants in Ischemic and Nonischemic Dilated Cardiomyopathy”**

Supplemental Methods

Variant Interpretation Criteria

Supplemental Table S1

Supplemental References

**Supplemental Methods**

*Exome Sequencing*

Genomic DNA was isolated from explanted heart tissue using the Macherey-Nagel NucleoSpin Tissue Mini Kit for DNA from Cells and Tissue (Düren, Nordrhein-Westfalen, Germany). DNA samples were assessed for concentration using a Qubit fluorometer (ThermoFisher Scientific, Waltham, MA) and for quality using the 4200 TapeStation (Agilent Technologies, Santa Clara, CA). Whole genome sequencing libraries were constructed using the xGen™ DNA Lib Prep EZ kit (Integrated DNA Technologies, Coralville, IA). Exome capture was performed using the xGen™ Exome Hyb Panel v2 kit (Integrated DNA Technologies) or the KAPA HyperPrep Kit (Roche). Libraries were quantified and qualified via Qubit and TapeStation. Multiplexed libraries were sequenced on a NovaSeq 6000 (Illumina, San Diego, CA) using 2x150 bp paired-end sequencing or a NovaSeq X Plus (Illumina, San Diego, CA) using 2x100 bp paired-end sequencing. Variant calling was performed using GATK-Haplotype Caller^1^ and Manta^2^ for indels.

*Gene Selection*

We assessed for variants in 36 genes associated with DCM as previously selected in the the DCM Precision Medicine Study ^3, 4^ with the addition of DMD given its association with DCM ^5^. The final 37 genes were: *ABCC9, ACTC1, ACTN2, ANKRD1, BAG3, CRYAB, CSRP3, DES, DMD, DSG2, DSP, EYA4, FLNC, ILK, JPH2, LAMA4, LDB3, LMNA, MYBPC3, MYH6, MYH7, MYPN, NEBL, NEXN, PDLIM3, PKP2, PLN, RBM20, SCN5A, SGCD, TCAP, TNNC1, TNNI3, TNNT2, TPM1, TTN, VCL.*

*Variant Filtering*

Variants were excluded if they were present in gnomAD exomes v4.1 with minor allele frequency ≥ 0.1% ^6, 7^ and if genotype quality was < 20.

*Variant Interpretation*

Variants were assigned as benign/likely benign or likely pathogenic/pathogenic if the variant had that classification in ClinVar (version June 30, 2024)^8^ and a review status of "reviewed_by_expert_panel" or "criteria_provided,_multiple_submitters,_no_conflicts." If no applicable ClinVar classification was present, variant interpretation was performed using the ClinGen guidelines for DCM ^9^ with modifications from the DCM Precision Medicine Study ^4^. A full specification of our variant interpretation criteria can be found in the Supplemental Material. Variant effect was determined using Ensembl Variant Effect Predictor ^10^ on Matched Annotation from the NCBI and EMBL-EBI (MANE) transcripts^11^. Proband counts were taken from submissions to ClinVar (version July 2024) with the phenotype of DCM. Final variant pathogenicity classification was based on the sum of points awarded from all criteria ^12^.

*Statistical Analysis*

Statistical analysis was performed in R (version 4.2.1, R Foundation for Statistical

Computing, Vienna, Austria).

**Variant Interpretation Criteria**

Variant interpretation criteria originated from Morales et al. ^9^ with updates from Jordan et al. and using the point system developed by Tavtigian et al.^12^.

Very strong evidence of pathogenicity

• PVS1, Null variant (nonsense, frameshift, canonical +/−1 or 2 splice sites, initiation
codon, single or multi-exon deletion) in LMNA/SCN5A

Strong evidence of pathogenicity

• PVS1_Strong, Null in FLNC/BAG3/TTN_A-band or exon with PSI > 0.9/DSP

• PS1, Same amino acid change as a previously established pathogenic variant
regardless of nucleotide change

• PS3, Well-established *in vitro* or *in vivo* functional studies supportive of a damaging effect on the gene or gene product

• PS4_Strong, Variant identified in ≥10 unrelated probands with consistent phenotypes

Moderate evidence of pathogenicity

• PVS1_Moderate, Null in VCL/PLN

• PM1, Located in RBM20 hotspot (exon 9, amino acids 634, 636, 637, 638)

• PM2, Absent from gnomAD or at extremely low frequency (<0.05%) in all gnomAD nonfounder populations ^9^

• PM4, Protein length changes due to stop-loss/in-frame deletions/insertions in a non-repeat region

• PM5, Novel missense change at an amino acid residue where a different missense change determined to be pathogenic has been seen before

• PS4_Moderate, Variant identified in ≥6 probands with consistent phenotypes

Supporting evidence of pathogenicity

• PP3, Multiple lines of computational evidence support a deleterious effect on the gene or gene product (REVEL >0.7)

• PS4_Supporting, Variant identified in ≥2 probands with consistent phenotypes

Stand alone evidence of benign impact

• BP1_Stantd_Alone, Missense variant in TTN

Strong evidence of benign impact

• BS1, Allele frequency is >0.05% in any gnomAD nonfounder population

• BS3, Well-established *in vitro* or *in vivo* functional studies shows no damaging effect on protein function or splicing

Supporting evidence of benign impact

• BP4, Multiple lines of computational evidence suggest no impact on gene or gene product (REVEL < 0.15)

• BP7, Unlikely to affect protein function based on calculated SO terms for the transcript of interest

Point system

| Strength | Pathogenic | Benign |
| --- | --- | --- |
| Indeterminate | 0 | 0 ^§^ |
| Supporting | 1 | −1 |
| Moderate | 2 | −2 ^†^ |
| Strong | 4 | −4 |
| Very Strong | 8 | −8 ^†^ |

| Category | Point ranges |
| --- | --- |
| Pathogenic | ≥ 10 |
| Likely Pathogenic | 6 – 9 ^¥^ |
| Uncertain | 0 – 5 |
| Likely Benign | −1 – −6 ^¥^ |
| Benign | ≤ −7 |

**Supplemental Table 1**

| **Variant^1^** | **F^2^ (x 10^-6^)** | **Type^3^** | **Gene** | **Coding** | **Protein** | **Evidence^4^** | **Class^5^** |
| --- | --- | --- | --- | --- | --- | --- | --- |
| chr1-156134838-C-T | NA | SG | LMNA | ENST00000368300  c.673C>T | ENSP00000357283  p.Arg225Ter | Clinvar/FC | P |
| chr2-178547890-TG-T | 0.68 | FS | TTN | ENST00000589042  c.93735del | ENSP00000467141  p.Val31247Ter | Clinvar/FC | LP |
| chr2-178548829-GTT-G | NA | FS | TTN | ENST00000589042  c.92795_92796del | ENSP00000467141  p.Lys30932ThrfsTer6 | NR | LP |
| chr2-178560772-GT-G | NA | FS | TTN | ENST00000589042  c.85359del | ENSP00000467141  p.Pro28454GlnfsTer7 | Clinvar/NFC | LP |
| chr2-178567508-TC-T | NA | FS | TTN | ENST00000589042  c.78623del | ENSP00000467141  p.Gly26208GlufsTer21 | NR | LP |
| chr2-178570990-ATTCT-A | NA | FS | TTN | ENST00000589042  c.75138_75141del | ENSP00000467141  p.Lys25046AsnfsTer8 | Clinvar/FC | P/LP |
| chr2-178577785-G-A | 1.37 | SG | TTN | ENST00000589042  c.68641C>T | ENSP00000467141  p.Arg22881Ter | Clinvar/FC | LP |
| chr2-178612453-CTCTTTTCCACAATG-C | NA | SG/FS | TTN | ENST00000589042  c.50058_50071del | ENSP00000467141  p.Tyr16686Ter | NR | LP |
| chr2-178738152-G-T | NA | SG | TTN | ENST00000589042  c.14301C>A | ENSP00000467141  p.Cys4767Ter | Clinvar/FC | LP |
| chr7-128844045-C-T | 2.05 | SG | FLNC | ENST00000325888  c.2971C>T | ENSP00000327145  p.Arg991Ter | Clinvar/FC | P/LP |
| chr10-110812298-G-A | 2.87 | MIS | RBM20 | ENST00000369519  c.1901G>A | ENSP00000358532  p.Arg634Gln | Clinvar/FC | P/LP |
| chr14-23425970-C-T | NA | MIS | MYH7 | ENST00000355349  c.2156G>A | ENSP00000347507  p.Arg719Gln | Clinvar/FC | P |
| chrX-32614303-CT-C | NA | FS/SPLICE | DMD | ENST00000357033  c.1481del | ENSP00000354923  p.Lys494ArgfsTer7 | Clinvar/NFC | LP |

**Footnotes/Abbreviations**: 1: Variants are described as chromosome-position-reference-alternate, with position provided per GRCh38; 2: F=minor allele frequency; 3: FS=frameshift, SG=stop gain, MIS=missense; 4: Clinvar/FC=variant found in ClinVar with fulfilled criteria for likely pathogenic/pathogenic status (which are detailed above in Variant Interpretation of Supplemental Methods), Clinvar/NFC=variant found in ClinVar but without fulfilled criteria for likely pathogenic/pathogenic status, NR=variant not found in ClinVar or previous literature; 5: LP=likely pathogenic, P=pathogenic.

**Supplemental References**

1. Van der Auwera GA, O'Connor BD. *Genomics in the Cloud: Using Docker, GATK, and WDL in Terra*: O'Reilly Media; 2020.

2. Chen X, Schulz-Trieglaff O, Shaw R, et al. Manta: rapid detection of structural variants and indels for germline and cancer sequencing applications. *Bioinformatics.* 2016;32:1220-1222.

3. Kinnamon DD, Morales A, Bowen DJ, Burke W, Hershberger RE, Consortium* DCM. Toward Genetics-Driven Early Intervention in Dilated Cardiomyopathy: Design and Implementation of the DCM Precision Medicine Study. *Circ Cardiovasc Genet.* 2017;10.

4. Jordan E, Kinnamon DD, Haas GJ, et al. Genetic Architecture of Dilated Cardiomyopathy in Individuals of African and European Ancestry. *JAMA.* 2023;330:432-441.

5. Johnson R, Otway R, Chin E, et al. DMD-Associated Dilated Cardiomyopathy: Genotypes, Phenotypes, and Phenocopies. *Circ Genom Precis Med.* 2023;16:421-430.

6. Pedersen BS, Brown JM, Dashnow H, et al. Effective variant filtering and expected candidate variant yield in studies of rare human disease. *NPJ Genom Med.* 2021;6:60.

7. Gudmundsson S, Singer-Berk M, Watts NA, et al. Variant interpretation using population databases: Lessons from gnomAD. *Hum Mutat.* 2022;43:1012-1030.

8. Landrum MJ, Chitipiralla S, Brown GR, et al. ClinVar: improvements to accessing data. *Nucleic Acids Res.* 2020;48:D835-D844.

9. Morales A, Kinnamon DD, Jordan E, et al. Variant Interpretation for Dilated Cardiomyopathy: Refinement of the American College of Medical Genetics and Genomics/ClinGen Guidelines for the DCM Precision Medicine Study. *Circ Genom Precis Med.* 2020;13:e002480.

10. McLaren W, Gil L, Hunt SE, et al. The Ensembl Variant Effect Predictor. *Genome Biol.* 2016;17:122.

11. Morales J, Pujar S, Loveland JE, et al. A joint NCBI and EMBL-EBI transcript set for clinical genomics and research. *Nature.* 2022;604:310-315.

12. Tavtigian SV, Harrison SM, Boucher KM, Biesecker LG. Fitting a naturally scaled point system to the ACMG/AMP variant classification guidelines. *Hum Mutat.* 2020;41:1734-1737.
